# Preterm birth leads to hyper-reactive cognitive control processing and poor white matter organization in adulthood

**DOI:** 10.1016/j.neuroimage.2017.11.055

**Published:** 2017-11-27

**Authors:** Alexander Olsen, Emily L. Dennis, Kari Anne I. Evensen, Ingrid Marie Husby Hollund, Gro C.C. Løhaugen, Paul M. Thompson, Ann-Mari Brubakk, Live Eikenes, Asta K. Håberg

**Affiliations:** a Department of Psychology, NTNU, Norwegian University of Science and Technology, Trondheim, Norway; b Department of Physical Medicine and Rehabilitation, St. Olavs Hospital, Trondheim University Hospital, Trondheim, Norway; c Department of Circulation and Medical Imaging, NTNU, Norwegian University of Science and Technology, Trondheim, Norway; d Imaging Genetics Center, Stevens Institute for Neuroimaging and Informatics, Keck School of Medicine, University of Southern California, Marina del Rey, CA, USA; e Department of Laboratory Medicine, Children’s and Women’s Health, NTNU, Norwegian University of Science and Technology, Trondheim, Norway; f Department of Public Health and Nursing, NTNU, Norwegian University of Science and Technology, Trondheim, Norway; g Department of Physiotherapy, Trondheim Municipality, Trondheim, Norway; h Department of Pediatrics, Sørlandet Hospital, Arendal Norway; i Department of Neuromedicine, NTNU, Norwegian University of Science and Technology, Trondheim, Norway; j Department of Medical Imaging, St Olavs Hospital, Trondheim University Hospital, Trondheim, Norway

**Keywords:** Executive function, Brain development, Psychiatric disorders, Intelligence, Magnetic resonance imaging

## Abstract

Individuals born preterm with very low birth weight (VLBW; birth weight ≤ 1500 g) are at high risk for perinatal brain injuries and deviant brain development, leading to increased chances of later cognitive, emotional, and behavioral problems. Here we investigated the neuronal underpinnings of both reactive and proactive cognitive control processes in adults with VLBW. We included 32 adults born preterm with VLBW (before 37th week of gestation) and 32 term-born controls (birth weight ≥10th percentile for gestational age) between 22 and 24 years of age that have been followed prospectively since birth. Participants performed a well-validated Not-X continuous performance test (CPT) adapted for use in a mixed block- and event-related fMRI protocol. BOLD fMRI and DTI data was acquired on a 3T scanner. Performance on the Not-X CPT was highly similar between groups. However, the VLBW group demonstrated hyper-reactive cognitive control processing and disrupted white matter organization. The hyper-reactive brain activation signature in VLBW adults was associated with lower gestational age, lower fluid intelligence score, and anxiety problems. Automated Multi-Atlas Tract Extraction (AutoMATE) analyses revealed that this disruption of normal brain function was accompanied by poorer white matter organization in the anterior thalamic radiation and the cingulum, as reflected in both reduced fractional anisotropy and increased mean diffusivity. These findings show that the preterm behavioral phenotype is associated with predominantly reactive-, rather than proactive cognitive control processing, as well as white matter abnormalities, that may underlie common difficulties that many preterm born individuals experience in everyday life.

## Introduction

Individuals born preterm with very low birth weight (VLBW; birth weight ≤1500 g) are at high risk of perinatal brain injuries and deviant brain development (Volpe, 2009), leading to increased chances of later cognitive, emotional, and behavioral problems (Johnson and Marlow, 2011; Lohaugen et al., 2010; Lund et al., 2012). Structural brain alterations and impaired neurodevelopmental outcomes are prevalent in the VLBW population, and persist into young adulthood (Eikenes et al., 2011;Nosarti et al., 2014; Rimol et al., 2016). However, findings regarding functional adaptations in the brain associated with preterm birth and VLBW are less clear. BOLD fMRI studies report both hyper- and hypo-activations in preterm and VLBW individuals, compared to term-born controls (Brittain et al., 2014; Froudist-Walsh et al., 2015; Griffiths et al., 2013; Kalpakidou et al., 2014; Salvan et al., 2014). Some studies report potential functional compensatory activations (Brittain et al., 2014; Froudist-Walsh et al., 2015), but others do not (Daamen et al., 2015). Various paradigms have been employed, but we lack a theoretical framework to account for the inconsistent findings from BOLD fMRI in individuals born preterm.

In the VLBW population, deficits in a broad range of cognitive domains are reported, and cognitive control dysfunction is prevalent (Bless et al., 2013; Lohaugen et al., 2010; Ostgard et al., 2016). Cognitive control processes in the brain operate on (at least) two different temporal scales (Braver, 2012; Dosenbach et al., 2008). Processes related to stable task-set maintenance (Dosenbach et al., 2008), or proactive cognitive control (Braver, 2012), are maintained over an extended period, typically across several trials in a cognitive task. Adaptive task control (Dosenbach et al., 2008), or reactive cognitive control (Braver, 2012), operates within a narrower time frame, and is associated with moment-to-moment processing and rapid adjustments based on particular stimuli.

In cognitively healthy individuals, a predominately reactive cognitive control brain activation signature is associated with lower fluid intelligence (Burgess and Braver, 2010) and anxiety (Fales et al., 2008), which both are prevalent in the VLBW population (Husby et al., 2016; Lohaugen et al., 2010; Lund et al., 2012). Moreover, preterm individuals primarily have problems with processes associated with a high demand on proactive cognitive control, whereas processes more closely related to adaptive task control are typically intact (Bless et al., 2013; Geldof et al., 2013; Pizzo et al., 2010). The “preterm behavioral phenotype” (Johnson and Marlow, 2011) may therefore be associated with predominantly reactive-, rather than proactive cognitive control processing.

Cognitive control functions depend on the interplay between frontoparietal brain regions. Disrupted white matter organization in cortico-cortical tracts has been related to cognitive control dysfunction in VLBW children and adults (Eikenes et al., 2011; Skranes et al., 2009). In addition to cortico-cortical connections, thalamo-cortical tracts are vulnerable to preterm birth (Ball et al., 2012, 2013; Volpe, 2009); reduced thalamo-cortical connectivity has been related to poorer cognitive function in preterm children (Ball et al., 2015). Here we focus on two key cortico-cortical and thalamo-cortical tracts; the cingulum (CGC) and the anterior thalamic radiation (ATR). These tracts are implicated in prior studies of white matter in preterm and VLBW individuals (Ball et al., 2013; Eikenes et al., 2011; Skranes et al., 2009), and connect key grey matter brain regions for both reactive adaptive task control and proactive stable task-set maintenance (Cooper et al., 2015; Metzler-Baddeley et al., 2012; Olsen et al., 2013).

Since 1986–88, a hospital-based cohort of VLBW individuals and a group of term-born controls has been followed since birth in Trondheim, Norway. This cohort has participated in several MRI studies investigating structural brain alterations (Eikenes et al., 2011; Skranes et al., 2007,^1997^, ^1993^). The present study is the first to report fMRI data from this cohort. A well-validated and controlled mixed block- and event-related fMRI design (Olsen et al., 2013,^2015^) was applied, using a novel theoretical framework in the context of preterm birth, and testing hypotheses about the role of hyper- and hypo activations, based on the temporal resolution of cognitive control processes (Braver, 2012; Dosenbach et al., 2008).

We hypothesized that (i) VLBW adults would demonstrate more reactive adaptive task control activations and less proactive stable task-set maintenance BOLD activations compared to term-born age- and sex-matched controls. We also predicted (ii) that this discrepancy in activation between VLBW adults and term-born controls would be accompanied by poorer organization as measured with diffusion tensor imaging (DTI) in cortico-cortical (CGC) and thalamo-cortical (ATR) white matter tracts selected *a priori*. Finally, (iii) the clinical and functional significance of VLBW-related BOLD and DTI alterations was evaluated by investigating their relationships to neonatal variables as well as clinical measures of cognitive control, fluid intelligence and anxiety problems.

## Materials and methods

### Participants

All participants were recruited through a cohort database from a hospital-based follow-up study (birth years 1986–88) in Trondheim, Norway, and have participated in several previous MRI studies at 1, 5, 14 and 20 years of age (Eikenes et al., 2011; Skranes et al., 2007,^1997^,^1993^). Of 54 VLBW individuals who were contacted from the original cohort, 18 (33%) did not consent. Of 48 controls contacted, two (4%) did not participate due to pregnancy, five (10%) could not participate because they had moved to a different part of the country, four did not consent (8%), and one (2%) that initially consented to the study did not undergo MRI. This left 36 young adults born preterm (before 37th week of gestation) with VLBW (birth weight ≤ 1500 g) and 36 term-born controls with normal birth-weight (≥10th percentile for gestational age) aged 22–24 years to be included in the present MRI study. As described elsewhere, this sample from the cohort did not differ in key variables from non-participants (Husby et al., 2013, 2016). Four VLBW adults were excluded from further MRI analysis; one due to failure to record behavioral data because of technical problems, one due to failure to comply to task instructions, one due to scanner technical problems, and one due to excessive head movements during fMRI acquisition. Four term-born controls were excluded from further analysis; one due to failure to record behavioral data because of technical problems, one due scanner technical problems, one due to excessive fMRI artifacts, and one due to excessive head movement during fMRI acquisition. This resulted in 32 VBLW adults (20 females, mean age = 22.51 years, SD = 0.69 years) and 32 term-born healthy controls (18 females, mean age = 22.70 years, SD = 0.62 years) in the final MRI sample. The study protocol was approved by the Regional Committee for Medical and Health Research Ethics in Central Norway (REK number 4.2005.2605) and was in accordance with the 1964 Helsinki declaration and its later amendments or comparable ethical standards. Written informed consent was obtained from all participants.

### Neonatal and demographic variables

Maternal age at birth, gestational age, birth weight, number of days on mechanical ventilator, number of days in the neonatal intensive care unit (NICU), and Apgar score (Apgar, 1953) at 1 and 5 min were included. Age and years of completed education at the time of fMRI/DTI were also included, in addition to parental socioeconomic status calculated according to Hollingshead’s Two factor index of social position (Hollingshead, 1958), recorded at 14 years of age. Independent samples *t*-test and Mann-Whitney U-tests were applied where appropriate for assessment of group differences, and p < 0.05 was considered statistically significant.

### Clinical measures of cognitive control function, fluid intelligence and anxiety

As a performance-based measure of cognitive control function, the Delis-Kaplan Executive Function System-Trail Making Test (D-KEFSTMT) letter-number switching (time to complete task) was used (Delis et al., 2005). The General Executive Composite (GEC) score from the Behavioral Rating Inventory of Executive Function-Adult version (BRIEF-A) was used as a self-report measure of cognitive control function (Roth et al., 2005). For both performance-based and self-reported cognitive control, raw scores were used for descriptive statistics, but these were inverted to allow for a more intuitive interpretation in regression analyses (higher score = better function). The Performance Index score from the Wechsler Adult Intelligence Scale III (WAIS-III; Pearson Assessment, USA) at 19 years of age was included as a measure of fluid intelligence. The Achenbach System of Empirically Based Assessment - Adult Self-Report (ASEBA-ASR) Anxiety Problems Scale was used in order to provide a measure of anxiety problems (Achenbach and Rescorla, 2003). Group differences were investigated using independent samples t-tests, and p < 0.05 was considered statistically significant.

### Design and procedure of fMRI task

The fMRI task used in this study was a Not-X continuous performance test (CPT) that has been validated in a large group of healthy individuals (Olsen et al., 2013) and tested in a clinical sample with chronic traumatic brain injury (Olsen et al., 2015). The task was inspired by the Conners’ CPT (Conners et al., 2003), but adapted and optimized for use in a mixed block- and event related fMRI paradigm (Olsen et al., 2013). The task is described in more detail elsewhere (Olsen et al., 2013, 2015), and in Supplementary Material 1.

A fiber-optic response device (ResponseGrip, Nordic NeuroLab, Bergen, Norway) was used for registration of responses. Participants were instructed to respond as quickly and accurately as possible by pressing a response button whenever a target (A-Z) was presented on the screen, and to withhold their response whenever the letter X appeared. Before MRI scanning, an experimenter ensured that participants performed the task as intended in a practice session outside the scanner room. Stimulus presentation and timing of stimuli was implemented using E-Prime 1.2 (Psychology Software Tools, Pittsburgh, USA). Stimuli were presented through MRI compatible video goggles (VisualSystem, Nordic NeuroLab, Bergen, Norway) or a head-coil-mounted mirror system and an MRI compatible monitor (Siemens AG, Erlangen, Germany). A relative difference of ~60 ms in stimuli onset between the goggles and monitor was detected through a quality control assessment using photodiodes and an oscilloscope. This was controlled for in the post-processing of the fMRI data.

### Analysis of Not-X CPT behavioral data

Based on behavioral raw data, the following CPT measures were calculated; Hit Reaction Time, Hit Reaction Time Standard Error, Omission Errors, and Commission Errors. To investigate change in performance as an effect of time-on-task (TOT) the task was divided into 4 time epochs after combining run 1 and 2. Each time epoch had identical length and was balanced with regards to task demands (Olsen et al., 2013). Difference scores (Δ) for each CPT measure was calculated by subtracting the score from the first quarter of the task from the last (Δ = time epoch 4-time epoch 1). To assess group differences, separate (for Not-X CPT and Δ Not-X CPT performance) 2 × 4 multivariate analyses of variances (MANOVA) were applied, with group as a fixed factor (VLBW adults, healthy controls) and the 4 performance measures as dependent variables.

### MRI acquisition

MRI data was acquired on a Siemens Trio scanner with Quantum gradients (30 mT/m) and a 12-channel Head Matrix Coil (Siemens AG, Erlangen, Germany). Foam pads around the participants’ heads were used to reduce movement. BOLD fMRI was acquired during Not-X CPT performance using a T2* echo-planar imaging pulse sequence (TR = 2400 ms, TE = 35 ms, FOV = 244 mm, matrix = 80 × 80, slice thickness = 3 mm, giving an in-plane resolution of 3 × 3mm, number of slices = 40). Before each BOLD sequence, 2 spin echo sequences (TR = 2010 ms, TE 35 ms, FOV = 244, slice thickness = 3 mm, matrix = 80 × 80, giving an in-plane resolution of 3 × 3mm) with opposite phase encoding (A-P and P-A) was acquired for correction of static magnetic field-induced distortion (Holland et al., 2010). The DTI sequence was a single-shot balanced-echo EPI sequence acquired in 30 non-collinear directions with b = 1000 s/mm2 (TR = 6800 ms, TE = 84 ms, slice thickness = 2.5 mm, matrix = 96 × 96, giving isotropic voxels of 2.5, number of slices = 55). For each slice, six images without diffusion weighting (b = 0), and 30 images with diffusion gradients were acquired. The DTI sequence was repeated twice to increase signal-to-noise ratio. A T1 MPRAGE volume was acquired for anatomical reference (TR = 2300 ms, TE = 30 ms, FOV = 256, slice thickness = 1.2 mm, matrix = 256 × 256, giving an in-plane resolution of 1 × 1mm).

### Preprocessing of fMRI data

Non-brain structures were removed with BET (Smith, 2002), motion corrected with MCFLIRT (Jenkinson et al., 2002), and geometrical distortions were corrected according to Holland et al. (2010). Data were smoothed (Gaussian kernel FWHM 6 mm), grand mean intensity normalized and high pass filtered (50s for block analyses and 25s for event related analyses). Each fMRI volume was linearly registered to their corresponding native high resolution T1 MPRAGE using 7 degrees of freedom (Jenkinson et al., 2002; Jenkinson and Smith, 2001). A transformation matrix was created by registration of the high resolution T1 MPRAGE to a 1 mm MNI standard template using 12 degrees of freedom and a warp resolution of 8 mm, and fMRI data was subsequently transformed into standard MNI space by applying this transformation matrix (Anderson et al., 2007a, b). BOLD activations were modeled by applying GLM. The hemodynamic response function was convolved using a standard Gamma variate. Contrasts were first computed separately for the two runs and then combined using a fixed-effects model. Mixed-effects models were used in subsequent analyses. The Stable task-set maintenance (STM; task block > fixation block) and the adaptive task control (ATC; non-targets > targets) contrasts included data from all time epoch of the task. To investigate TOT effects, the following contrasts were modeled; STM TOT increase (task block time epoch 4 > task block time epoch 1), STM TOT decrease (task block time epoch 1 < task block time epoch 4), ATC TOT increase (non-targets time epoch 4 > non-targets time epoch 1), and ATC TOT decrease (non-targets time epoch 1 > non-targets time epoch 4).

### Whole brain and ROI fMRI analyses

First, an omnibus whole-brain analysis was performed to reveal main differences between VLBW adults and controls regarding STM, ATC, and corresponding TOT effects. SPMs were corrected for multiple comparisons by using a cluster threshold of Z > 2.3, and a corrected cluster significance of P < 0.05. Main peak Z-values with up to 5 local maxima and size of clusters (number of voxels) in standard 1 × 1×1mm MNI space were extracted. Anatomical denotation was determined by using the Harvard Oxford cortical and subcortical structural brain atlases as incorporated in the FSL software and visual inspection. The main fMRI analyses revealed two Regions of interest (ROIs) for adaptive task control, named ATC ROI-1 and ATC ROI-2, and two for stable task-set maintenance, named STM ROI-1 and STM ROI-2, respectively (see Results and Table 3). Post-hoc ROI analyses were performed to further explore between-group effects in key activation areas across birthweight, GA, as well as associations with DTI measures, cognitive control function, fluid intelligence and anxiety problems. For this purpose, normalized average parameter estimates from voxels included in the clusters that demonstrated statistically significant results for the main contrasts in the whole brain analysis was extracted from each individual and used.

Both the clinical and functional significance of the fMRI results were investigated. Associations between Not-X CPT task performance and fMRI parameter estimates were evaluated in separate partial correlation models (for VLBW and Controls) adjusting for birthweight and GA. It is crucial for the validity of the interpretation of BOLD activation alterations that fMRI task performance is kept highly similar and/or adjusted for across groups or conditions (Price et al., 2006). Consequently, analyses exploring the external validity of fMRI activations were adjusted for online fMRI task performance. The independent within-group effects of birth weight and GA were evaluated in separate partial correlation models for VLBW adults and controls investigating associations between fMRI data and birth weight (adjusted for GA and Not-X CPT performance) as well as associations between fMRI data and GA (adjusted for birth weight and Not-X CPT performance). The fMRI findings were also related to fluid intelligence, anxiety problems, as well as performance-based and self-reported cognitive control function in separate partial correlation models adjusted for Not-X CPT performance, birthweight and GA.

### DTI tract average and element-wise analyses

AutoMATE (automated multi-atlas tract extraction) was used for automated tract extraction, and is described fully in prior papers (Jin et al., 2013, 2014, 2012), and in Supplementary Material 2. We focused our further analyses on the a-priori tracts ATR and CGC. AutoMATE yields text files with dMRI measures extracted along tract. For each streamline, we sample FA at 15 points along-tract. There were 333 streamlines in the bilateral ATR, and 217 for the bilateral CGC. Extracting fractional anisotropy (FA) and mean diffusivity (MD) along tract gave us 333 × 15 data points for the ATR and 217 × 15 data points for the CGC. We examined FA and MD element-wise (considering each of the 333 × 15 and 217 × 15 data points) and averaged within each tract. Multiple linear regressions testing for group differences in tract averages, co-varying for age and sex were performed. The analyses were corrected for multiple comparisons using FDR (q < 0.05) (Benjamini and Hochberg, 1995) across fractional FA and MD, as well as across the bilateral ATR and bilateral CGC. We also ran within-group (VLBW and controls) regressions testing for associations between DTI measures (averaged within each of the four tracts) and neonatal factors (GA and birth weight), performance-based cognitive control function, self-reported cognitive control function, fluid intelligence, and anxiety, co-varying for age and sex. Associations with GA and birth weight were tested separately, by adjusting for the other. Analyses were corrected for multiple comparisons across all analyses within each group using FDR (q < 0.05). We ran the regressions listed above on the element-wise estimates of FA and MD correcting for multiple comparisons across all points and co-varying for age and sex, as described above (*q* < 0.05).

### Associations between fMRI and DTI

A post-hoc analysis was performed to evaluate within-group (VLBW and controls) associations between DTI measures (FA and MD) and ATC ROI-1, ATC ROI-2, STM ROI-1, and STM ROI-2 BOLD activation. Both tract-average and element-wise analyses were performed separately in the VLBW and healthy control groups, co-varying for age and sex.

## Results

### Neonatal and demographic data

VLBW adults had significantly lower birth weight, gestational age, and Apgar scores after 1 and 5 min, compared to controls (Table 1). VLBW adults also spent more days in the NICU and on mechanical ventilator than controls (Table 1). There were no statistically significant differences between the groups in age, sex, education, parental socioeconomic status at 14 years, or maternal age at birth (Table 1).

### Clinical measures of cognitive control function, fluid intelligence and anxiety

The VLBW group had poorer performance-based and self-reported cognitive control function compared to controls as measured with standard clinical instruments (Table 1). Moreover, VLBW adults exhibited a lower fluid intelligence score than controls (Table 1). There was no statistically significant difference in self-reported anxiety problems between VLBW adults and controls (Table 1).

### Not-X CPT behavioral data

There were no multivariate or univariate statistically significant group differences between VLBW adults and controls for overall performance *(Not X-CPT performance)* or time-on-task effects (Δ *Not X-CPT performance)* (Table 2).

### Results of fMRI analyses

The STM contrast revealed greater activation in controls relative to VLBW adults (Fig. 3 and Supplementary Video 1). Two statistically significant clusters were found; one encompassed regions bilaterally in the frontal pole and the anterior cingulate gyrus (STM ROI-1), the other included regions in the posterior cingulate gyrus and precuneus (STM ROI-2) (Fig. 3 and Supplementary Video 1). For the ATC contrast an opposite pattern was found, with greater activation in VLBW adults as compared to controls (Fig. 3 and Supplementary Video 1). Two clusters were found; one encompassed regions in the posterior cingulate gyrus and precuneus (ATC ROI-1), and another included regions in the right lateral occipital cortex and angular gyrus (ATC ROI-2). The activation related to STM and ATC in posterior brain regions had minimal anatomical overlap (Fig. 3 and Supplementary Video 1). No regions showed increased activations in VLBW relative to controls for the STM contrast. Likewise, there were no regions with increased activation in controls vs. VLBW adults for the ATC contrast. More detailed information on cluster size and locations are presented in Table 3. There were no between-group differences for STM TOT. Controls had a higher relative increase of activation for ATC TOT compared to VLBW adults, in regions encompassing the right lateral occipital cortex, fusiform gyrus, inferior and middle temporal gyrus (main peak MNI coordinates: x = 55, y = −49, z = −9, middle temporal gyrus, size = 5449 voxels, Z = 3.81).

**Fig. 1.**
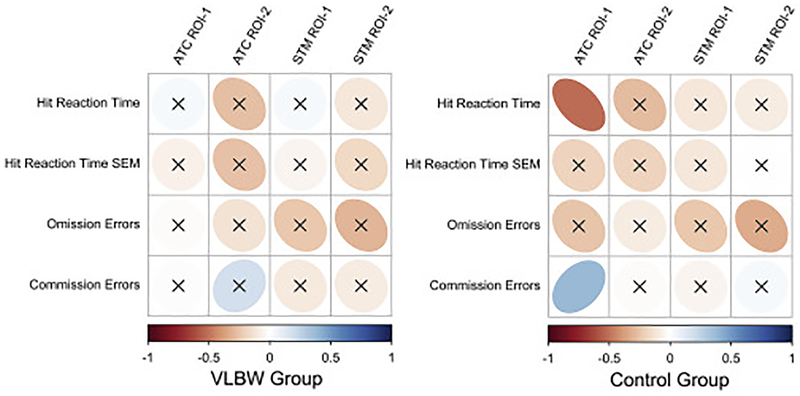
Within-group associations between BOLD activation from ROIs and online fMRI (Not-X-CPT) task performance. Correllogram displaying within-group partial correlations (adjusted for GA and birth weight). Positive partial correlations are displayed in blue, and negative partial correlations are displayed in red. Color intensity and shape of the ellipses are proportional to the correlation coefficients. Non-significant (p > 0.05) correlation coefficients are indicated with an “X” over the ellipse. SEM = Standard error of the mean. ATC = Adaptive task control. STM = Stable task-set maintenance. ROI = Region of interest.

**Fig. 2.**
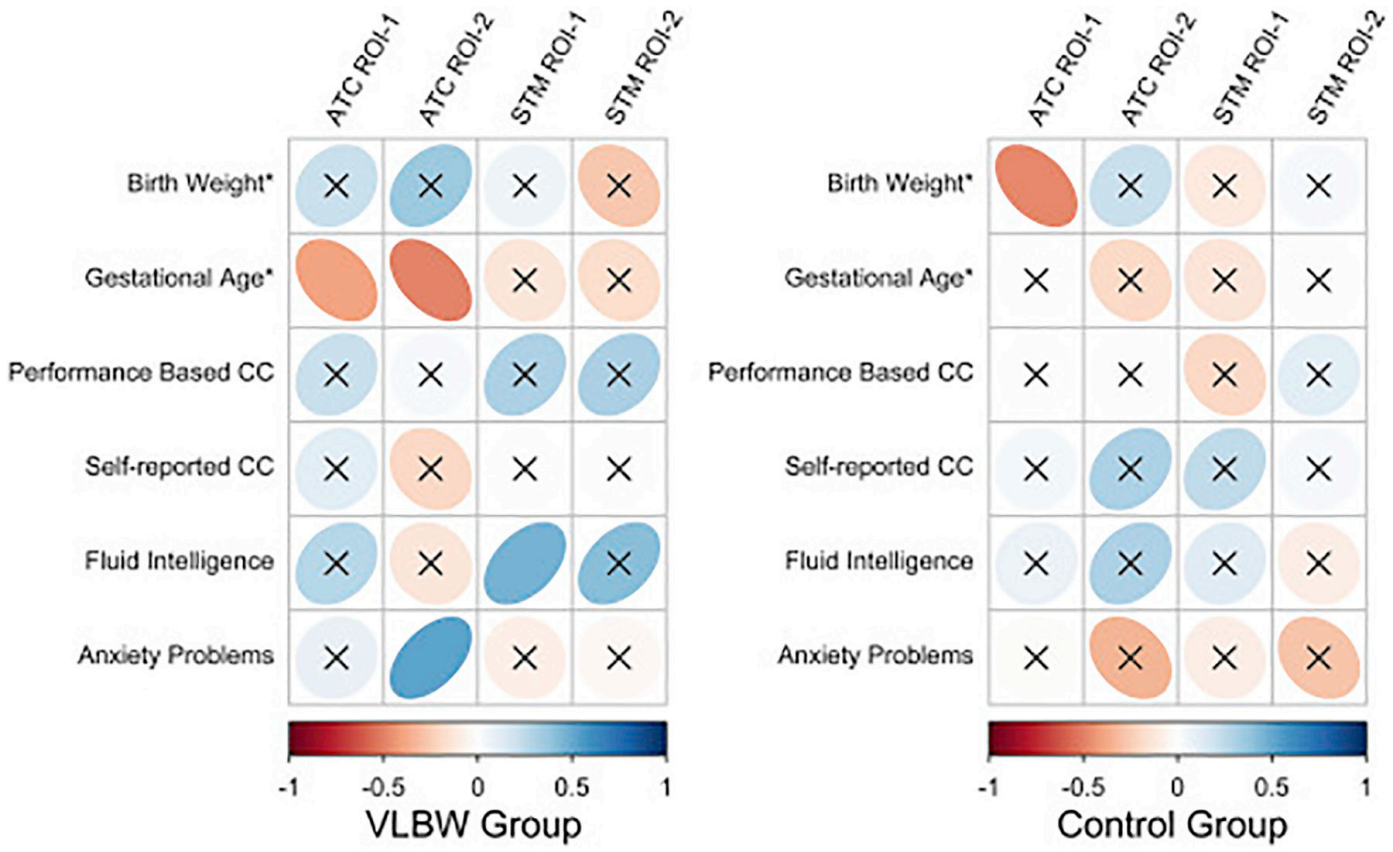
Within-group associations between BOLD activation from ROIs and birth weight, gestational age, as well as behavioral measures. Correllogram displaying within-group partial correlations (adjusted for GA, birth weight and fMRI task performance).*=associations with GA and BW were evaluated separately, by adjusting for the other. Positive partial correlations are displayed in blue, and negative partial correlations are displayed in red. Color intensity and shape of the ellipses are proportional to the correlation coefficients. Non-significant (p > 0.05) correlation coefficients are indicated with an “X” over the ellipse. ATC = Adaptive task control. STM = Stable task-set maintenance. ROI = Region of interest. CC = Cognitive control. For performance-based and self-reported CC, raw scores were inverted to allow for more intuitive interpretation (higher score = better function).

There were no statistically significant associations between BOLD activation in the various ROIs and on-line Not-X CPT performance for the VLBW group (Fig. 1). In the control group, higher ATC ROI-1 activation was associated with reduced Hit RT (r = −0.51, p = 0.004), and more commission errors (r = 0.38, p = 0.038) (Fig. 1). The within-group clinical and functional significance of BOLD alterations was investigated by testing associations with birth weight, GA, fluid intelligence, performance-based and self-reported cognitive control function, as well as anxiety problems (Fig. 2). In the VLBW group, ATC ROI-1 and ROI-2 activation was negatively associated with GA, and ATC ROI-1 activation was positively associated with anxiety problems. The VLBW group also exhibited a statistically significant positive association between STM ROI-1 activation and fluid intelligence. In the control group, the only statistically significant result was a negative association between ATC ROI-1 activation and birth weight.

### Results from DTi analyses

There were statistically significant group differences in FA and MD in the bilateral ATR and CGC, with lower average FA and higher average MD across these tracts in VLBW adults compared to controls (Table 4). In the element-wise comparison (examining FA and MD along tract) between VLBW adults and controls, we express the results as percentages, indicating the number of points showing significant group differences relative to the number of data points for that tract. For example, the left ATR contained 171 streamlines that were sampled at 15 points along each streamline, for a total of 2565 data points. *P*-values below the FDR threshold in 192 points would mean 7.5% of the tract showed significant group differences. The percentages of the tract showing significant group differences in FA were: left ATR: 7.5%, right ATR: 7.2%, left CGC: 28.5%, right CGC: 10.8%. For MD, the percentages were: left ATR: 1.5%, right ATR: 4.6%, left CGC: 3.4%, right CGC: 7.2%. The element-wise FA and MD results from the group analysis, including anatomical information, are shown in Fig. 4 and Supplementary Video 2.

Next, we examined within-group effects of GA, birth weight, and behavioral measures on tract averages. There were no statistically significant associations in the term-born control group. In the VLBW group, birth weight was positively associated with FA in the left CGC, worse self-reported cognitive control function associated with higher MD in the left CGC, fluid intelligence was positively associated with FA bilaterally in the CGC, and worse performance-based cognitive control function was associated with lower FA bilaterally in the CGC. There were no statistically significant associations between any of the DTI measures and GA or anxiety problems. These results are shown in Table 5. Examining element-wise measures in the VLBW group, we found clusters associated with birth weight, as well as self-reported and performance-based cognitive control function (Table 6). Birth weight was positively associated with FA in the left CGC, worse self-reported cognitive control function was associated with higher MD in the right CGC, worse performance-based cognitive control function was associated with lower FA and higher MD in both the ATR and CGC (Fig. 5). There were no significant associations between these measures and FA or MD along tracts in the control group.

### Within-group associations between fMRI ROI activations and DTI

Associations between fMRI ROI activations and DTI variables were examined in a *post hoc* analysis. For the tract-average analysis, the only statistically significant finding was an association between higher BOLD activation in the STM ROI-2 and higher average FA in the left ATR (*t* = 4.4, p = 0.00018) in the control group. There were no statistically significant associations within the VLBW group. For the element-wise analysis there were no statistically significant associations with fMRI ROI activations in either of the groups. A rather strict control for multiple comparisons (see method section for details) was applied throughout all analyses to minimize the chance of Type-I errors. Given the lack of other studies investigating associations between brain function and structure in adults born with VLBW, results uncorrected for multiple comparisons are included in Supplementary Material 3 (tract average results) and 4 (element-wise results) as this may be helpful when planning future studies focusing on replication or meta-analysis (Lieberman and Cunningham, 2009).

## Discussion

The main finding in this study was that adults born preterm with VLBW exhibited altered hemodynamic responses associated with predominantly reactive-, rather than proactive cognitive control processing. This hyper-reactivity was accompanied with poorer white matter organization in the CGC and ATR, and was associated with lower gestational age, lower fluid intelligence, and anxiety problems. These findings support the view that the preterm behavioral phenotype is associated with hyper-reactive cognitive control processing and white matter abnormalities, that may underlie common difficulties that many preterm-born individuals experience in everyday life. In other words, we here present evidence that suboptimal organization of the central nervous system due to the consequences of preterm birth with VLBW leads to a dysfunctional hyper-reactive brain state that extends into adulthood.

By utilizing a novel theoretical framework based on the temporal resolution of cognitive control processes (Braver, 2012; Dosenbach et al., 2008; Olsen et al., 2013, 2015), we here account for both hyper- and hypo- BOLD activations observed in VLBW individuals. VLBW adults had more reactive adaptive task control activations, and less proactive stable task-set maintenance activations compared to controls. This demonstrates a dysregulation in the balance between the proactive and reactive brain systems. Importantly, there were no statistically significant group differences in fMRI task performance (Price et al., 2006), but VLBW individuals had lower performance-based and self-reported cognitive control function, as well as a lower fluid intelligence score on standard clinical measures. A well-regulated balance between proactively planning behavior and being able to quickly react to sudden stimuli is crucial for adaptive functioning. During performance of interference tasks, cognitively healthy individuals rely more on proactive cognitive control processes during high expectancy conditions, and more on reactive cognitive control processes during low expectancy conditions (Burgess and Braver, 2010). Accordingly, our results indicate that VLBW adults experience the world in a state of increased vigilance to low-frequent events. This provides novel insight to prior reports that VLBW individuals have excessive attention towards irrelevant stimuli/dis-tractors (Aasen et al., 2016; van de Weijer-Bergsma et al., 2008). Moreover, the fact that VLBW individuals displayed a predominantly hyper-reactive brain activation signature, is in line with prior studies reporting that they have problems with top-down modulation of cognitive control processes (Bless et al., 2013; Geldof et al., 2013; Pizzo et al., 2010), while bottom-up processes such as altering and the orienting response often remain intact (Geldof et al., 2013; Pizzo et al., 2010). Facilitation of performance through top-down processes is believed to be associated with successful implementation of perceptual, motor- and attentional task-sets, and is linked to neural activation within a fronto-parietal system (Corbetta and Shulman, 2002). VLBW individuals in our study had notable hypo-activations associated with proactive STM in fronto-parietal regions overlapping with this top-down system. We suggest that a failure to properly engage this system and implement and maintain appropriate task-sets can explain the shift towards relying more on the reactive system.

Within the VLBW group there was a negative association between ATC activation and GA, meaning that being born more preterm was associated with increased hyper-reactivity. This indicate that pre- and neonatal factors are relevant for the development of a more reactive adaptive task control. When adjusting for birthweight and GA, lower STM activation in the VLBW group was associated with lower fluid intelligence, and higher ATC activation was associated with more anxiety problems (Fig. 2). This is in line with prior studies on anxiety and fluid intelligence in cognitively healthy individuals (Burgess and Braver, 2010; Fales et al., 2008), and demonstrate that the observed BOLD alterations in the VLBW adults indeed have functional and clinical significance. In this study, we used the Performance Index score of the WAIS-III to measure fluid intelligence. This may be considered a broader measure of fluid intelligence than the Raven’s Advanced Progressive Matrices, which was previously linked to a proactive cognitive control pattern in healthy controls (Burgess and Braver, 2010). This difference may account for the lack of finding such an association within the healthy controls in this study.

**Fig. 3.**
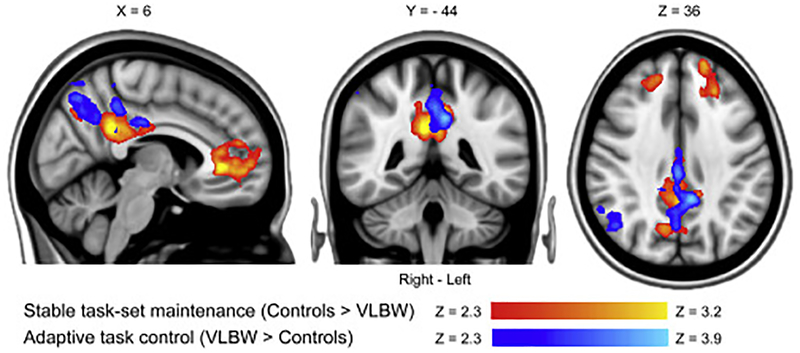
Comparison of stable task-set maintenance and adaptive task control BOLD activations during Not-X CPT performance across VLBW adults and controls. The stable task-set maintenance (STM) contrast revealed increased activations in controls relative to VLBW adults, with two statistically significant clusters; one encompassing regions bilaterally in the frontal pole and the anterior cingulate gyrus (STM ROI-1), and another including regions in the posterior cingulate gyrus and precuneus (STM ROI-2). For the Adaptive task control contrast (ATC), an opposite pattern was evident, with increased activations in VLBW adults as compared to controls, also with two statistically significant clusters; one encompassing regions in the posterior cingulate gyrus and precuneus (ATC ROI-1), and another including regions in the right lateral occipital cortex and angular gyrus (ATC ROI-2). See Table 3 for details on location of main peaks, local maxima, and size of significant clusters. Results were achieved using a mixed effects model corrected for multiple comparisons using a cluster threshold of Z > 2.3 and a corrected cluster significance threshold of p < 0.05. Results are presented on a 1-mm MNI standard space template. VLBW = Very low birth weight (≤1500 g). MNI = Montreal Neurological Institute. ROI = Region of Interest. See Supplementary Video 1 for a more detailed presentation of these results.

**Fig. 4.**
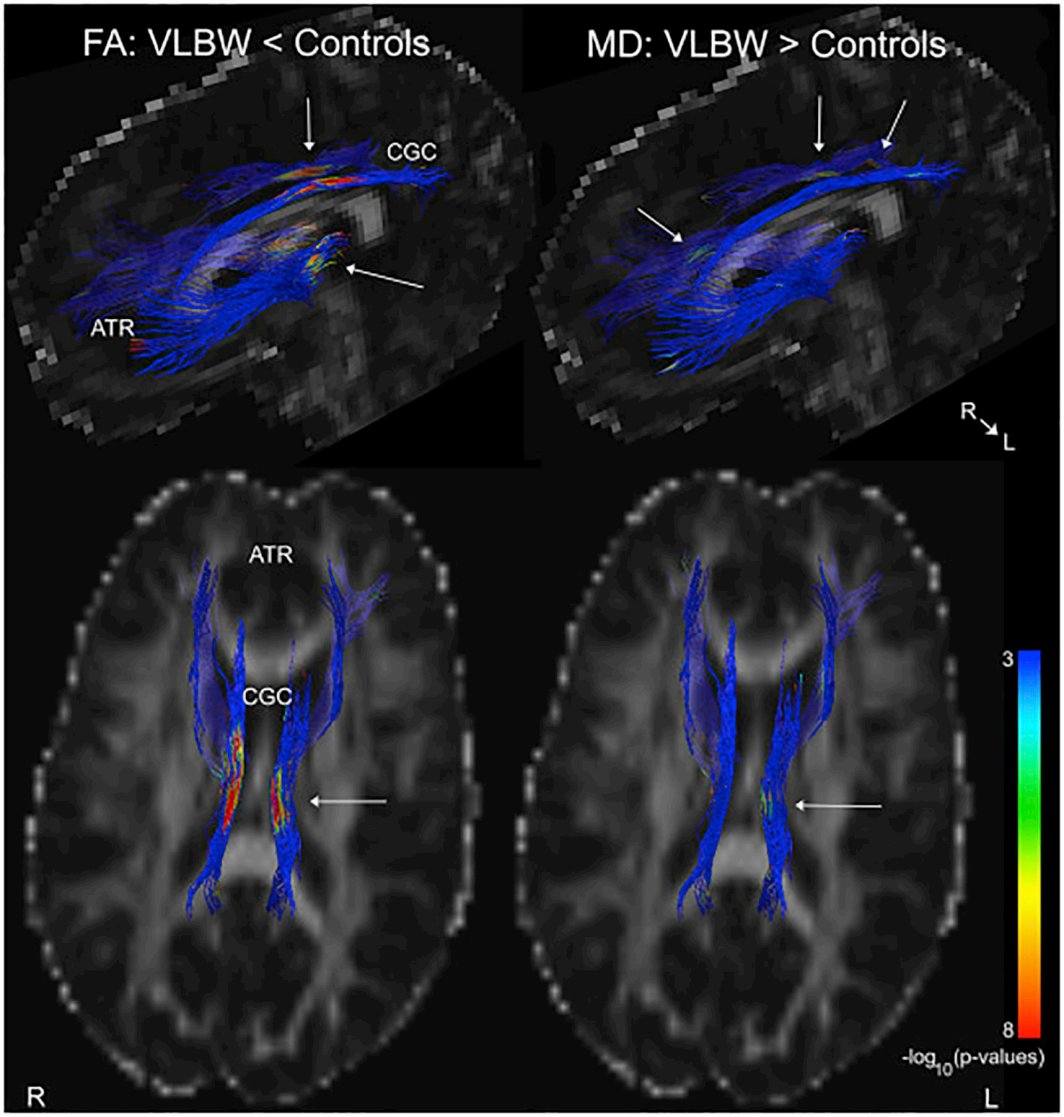
FA and MD group differences between VLBW adults and controls. Diffusion tensor imaging (DTI) data were analyzed using Automated Multi-Atlas Tract Extraction (AutoMATE, see Jin et al., 2012; Jin et al. 2013, 2014 for details) focusing on the cingulum (CGC) and anterior thalamic radiation (ATR). Results shown are from a multiple linear regression analysis testing for element-wise group differences in FA and MD, co-varying for age and sex. Results were corrected for multiple comparisons using FDR (q < 0.05). Above is a thresholded p-map, with blue areas representing areas at or above the FDR threshold, and not significantly different between groups. Areas that are green-red are those with increasingly significant p-values, as indicated in the color bar, where the VLBW group had lower FA and higher MD than controls. The color bar indicates the −log10(p-value). See Supplementary Video 2 for a more detailed presentation of these results.

**Fig. 5.**
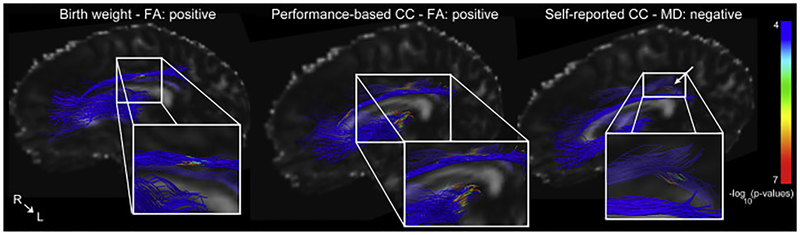
Element-wise associations with clinical and behavioral variables. Element-wise within-group associations between FA/MD, and clinical/behavioral variables. Results are shown from a multiple linear regression testing for element-wise effects, co-varying for age and sex, within the VLBW group. Only results that were statistically significant are shown. Results were corrected for multiple comparisons using FDR (q < 0.05). This is a thresholded p-map, with blue areas representing no significant correlation. Areas that are green-red are those showing significant correlations. The directions of correlations are indicated as ‘positive’ or ‘negative’. The color bar indicates the −log10 (p-value). For performance-based and self-reported CC, raw scores were inverted to allow for more intuitive interpretation (higher score = better function).

Two clusters of STM hypo-activation were evident in the VLBW group; one encompassed regions bilaterally in the frontal pole and the anterior cingulate gyrus, and the other included the posterior cingulate gyrus and precuneus (Fig. 3). The two regions of STM hypo-activation overlap to a large degree with regions exhibiting decreased BOLD activation associated with attention allocation in young adults born preterm (Lawrence et al., 2009), and with increasing working memory/cognitive control load in extremely preterm born children (Griffiths et al., 2013). Moreover, lower activation in brain regions encompassing the anterior cingulate cortex has been found in children with attention-deficit hyper activity disorder (Liotti et al., 2005) and autism spectrum disorders (Agam et al., 2010), which are prevalent conditions in preterm individuals. ATC activation in VLBW individuals was increased in posterior brain regions (Fig. 3). Prior studies have shown increases in BOLD activation in young adults born preterm in posterior brain regions in association with motor response inhibition (Lawrence et al., 2009; Nosarti et al., 2006). Increased posterior brain activation may both indicate a sign of immature brain development, as well as a compensatory mechanism. Children rely more on posterior brain regions during response inhibition than adults (Booth et al., 2003), and there is also evidence that posterior brain regions may play a more general compensatory role, when other more task specific brain regions are not fully matured (Durston and Casey, 2006). However, the existing evidence for compensatory BOLD activations in individuals born preterm is not straightforward, with some studies demonstrating potential functionally compensatory activations (Brittain et al., 2014; Froudist-Walsh et al., 2015), and others not (Daamen et al., 2015). Despite the high level of neuroplasticity in the newborn (Hensch, 2004; Kleim and Jones, 2008), the potential for functional compensatory adaptations may be lower after pre-/perinatal injuries than in adults with acquired brain damage. As many functional networks have yet to be developed in the newborn (Doria et al., 2010), accumulative developmental problems may rather become the result of an initial injury or dysfunction (van de Weijer-Bergsma et al., 2008; Woodward et al., 2005). In adults with chronic moderate-to-severe traumatic brain injury, the ability to increasingly engage more proactive cognitive control processes is associated with less self-reported cognitive control problems in everyday situations (Olsen et al., 2015). In the present study, however, there were no STM TOT differences between VLBW adults and the control group. Rather, the controls had more pronounced ATC TOT activations in posterior brain regions compared to VLBW adults. In context of the main finding, this means that controls increased the demand on reactive ATC as the task progressed, while VLBW individuals had a constant hyper-reactivity in this system. Although the generally increased ATC activation in VLBW individuals may represent some sort of compensatory mechanism because of the reduced ability to proactively maintain task-sets, it is at best suboptimal; it was not dynamically adjusted throughout the task and was associated with more anxiety problems rather than better everyday cognitive control function.

The VLBW group had poorer white matter organization in the ATR and CGC. This is a typical finding in VLBW individuals at different ages, also in prior studies from our cohort (Eikenes et al., 2011; Skranes et al., 2007, 1997, 1993). Poorer white-matter organization in terms of lower FA and higher MD, can be caused by factors such as reduced myelination, demyelination, dysmyelination, fewer axons, and poor axonal packing (Beaulieu, 2002; Song et al., 2005), and may reflect the increased risk of disturbed maturation of oligodendrocytes, as well as injury of axons and sub plate neurons after being born preterm with VLBW (Volpe, 2009). Confirming the important role of the CGC and ATR for cognitive function in general, and cognitive control function in particular (Ball et al., 2015; Cooper et al., 2015; Metzler-Baddeley et al., 2012), VLBW individuals had several statistically significant associations between DTI measures and standard clinical measures. Worse performance-based cognitive control function and fluid intelligence was associated with lower FA, and more self-reported cognitive control problems were associated with increased MD. For the tract-average measures, these associations were primarily found in the cingulum, which is in line with findings from VLBW adolescents (Skranes et al., 2009). The element-wise analyses extended this finding, and demonstrated that worse performance-based cognitive control was associated with lower FA and higher MD in both the CGC and ATR. There were no statistically significant associations between FA/MD and behavioral measures within the controls. Thalamo-cortical projections are developed and enter the cortex between 24 and 32 weeks of GA, whereas cortico-cortical axons enter the cortex slightly later between 32 and 36 weeks of GA (Kostovic and Jovanov-Milosevic, 2006), and are therefore susceptible to potential detrimental effects of preterm birth (Volpe, 2009). Altered thalamo-cortical connectivity has been found already at term for preterm born individuals (Ball et al., 2013), and explain as much as 11% of the variance in cognitive function in two-year olds (Ball et al., 2015). Prior studies on preterm birth and VLBW have shown that white matter organization, especially in longer cortico-cortical association tracts, is also affected in late childhood and adolescence (Skranes et al., 2009; Vang-berg et al., 2006), as well as into young adulthood (Eikenes et al., 2011). The present study demonstrates clearly that alterations in both thalamo-cortical and cortico-cortical tracts due to preterm birth and VLBW persist into adulthood, and have functional and clinical consequences.

The only statistically significant finding linking fMRI and DTI measures was found in the control group where there was an association between higher STM activation in posterior brain regions (posterior cingulate cortex/precuneus) and higher average FA in the right ATR. This indicates that STM activation may depend on input from the thalamus to the prefrontal cortex. The limited associations between fMRI and DTI measures may also suggest that the present study was underpowered with regards to detecting such effects. Due to this, and few prior VLBW studies integrating fMRI and DTI, we have included results uncontrolled for multiple comparisons as supplementary material. These exploratory results support the importance of the ATR for STM activation. This was evident from associations between higher tract-average FA in the ATR and higher STM activation within the frontal cortex in both VLBW individuals and controls. The only association for ATC activation was found in relation to lower MD in the right ATR in controls. The element-wise analysis also pointed in the same direction as those based on tract averages, but these also revealed additional similar associations within the cingulum (Supplementary Material 4). A study investigating a highly-selected group with perinatal brain injury (Froudist-Walsh et al., 2015) has previously demonstrated a negative association between cingulum tract volume and BOLD activation in the bilateral perisylvian cortex. However, as the finding in a similar region did not survive when controlling for multiple comparisons in the present study, the role of the cingulum for STM processing should be interpreted with caution. Another study has shown that young adults born very preterm had reduced FA in tracts passing through a thalamic region where they had different memory related BOLD activation than controls (Salvan et al., 2014). However, the same study did not report any results on association between the actual level of BOLD activation and white matter micro-structure. Although uncorrected results in the present study demonstrated possibly interesting findings, future studies including larger samples should be performed to further ascertain the presence of associations between brain function and white matter microstructure in VLBW individuals.

In conclusion, we found evidence that young adults born preterm with VLBW exhibit hyper-reactive cognitive control processing and disrupted brain microstructure, that may underlie common difficulties that these individuals experience in everyday life. The consequences of preterm birth with VLBW seems to leave the brain in a hyper-reactive state extending into adulthood. Future studies should aim to replicate this finding in other cohorts, and investigate how this dysfunctional brain alteration may be remediated by pharmacological or behavioral interventions.

## Supplementary Material

Supplementary Video 1

Supplementary Video 2

Supplementary data

## Figures and Tables

**Table 1:** Neonatal data, behavioral measures, and demographics.

	VLBW (n = 32, 20 female)			Control (n = 32, 18 female)			Group difference statistics	
	n	Mean/Median	SD/min-max	n	Mean/Median	SD/min-max	P	Effect size
Maternal age (years)^a^	32	28.45	5.38	32	30.43	4.70	0.122	*ηρ*^2^ = 0.039
Birth weight (grams)^a^	32	1214.84	246.31	32	3651.56	361.15	<0.001	*ηρ*^2^ = 0.939
Gestational age (weeks)^a^	32	29.28	2.61	32	39.47	1.14	<0.001	*ηρ*^2^ = 0.863
Apgar score after 1 min^b^	32	7	1–8	29	9	7–9	<0.001	*r* = 0.672
Apgar score after 5 min^b^	32	9	1–9	30	10	1–10	<0.001	*r* = 0.624
Days in NICU^b^	31	61	25–386	32	0	0–9	<0.001	*r* = 0.915
Days on ventilator^b^	32	1	0–44	32	0	0–0	<0.001	*r* = 0.603
Parental SES (at 14 years)^b^	32	4	1–5	30	3	2–5	0.288	*r* = 0.135
Age at fMRI/DTI^a^	32	22.51	0.69	32	22.70	0.62	0.236	*ηρ*^2^ = 0.021
Years of education^a^	31	11.70	1.10	30	11.93	1.23	0.457	*ηρ*^2^ = 0.009
Performance-based CC^a^	32	106.34	22.87	32	95.16	17.12	0.004	*ηρ*^2^ = 0.128
Self-reported CC^a^	31	84.47	36.71	32	62.75	18.31	0.030	*ηρ*^2^ = 0.073
Fluid Intelligence^a^	26	89.58	15.42	24	104.54	10.35	<0.001	*ηρ*^2^ = 0.249
Anxiety Problems^a^	31	3.42	3.08	32	2.53	2.23	0.194	*ηρ*^2^ = 0.027

SES = Socio Economic Status, SD = Standard Deviation. VLBW = Very Low Birth Weight (≤1500 g). CC = Cognitive Control. Group differences were investigated using

a Independent Samples T-Test, or

b Mann Whitney *U* Test where appropriate.

**Table 2:** Not X-CPT and *Δ* Not X-CPT measures.

Variable	MANOVA	Group	n	Mean	95% CI of means	95% CI of difference	P	*ηρ*^2^
Not X-CPT								
Hit RT (msec)	F (4, 59) = 0.421, p = 0.793, *ηρ*^2^ = 0.028	VLBW	32	424.68	400.68, 448.69	− 21.82, 45.01	0.485	0.008
		Control	32	412.76	388.75, 436.76			
Hit RT SEM		VLBW	32	7.52	6.22, 8.81	− 0.78, 2.82	0.284	0.019
		Control	32	6.53	5.23, 7.82			
Omissions		VLBW	32	11.19	5.51, 16.87	− 7.59, 8.27	0.994	<0.001
		Control	32	11.16	5.48, 16.83			
Commissions		VLBW	32	16.03	12.67, 19.40	− 4.65, 4.71	0.990	<0.001
		Control	32	16.00	12.64, 19.36			
*Δ* Not X-CPT								
*Δ* Hit RT (msec)	F (4, 59) = 1.400, p = 0.245, *ηρ*^2^ = 0.087	VLBW	32	11.39	− 3.47, 26.26	− 29.21, 12.84	0.440	0.010
		Control	32	19.57	4.71, 34.44 *			
*Δ* Hit RT SEM		VLBW	32	3.69	0.93, 7.29 *	− 2.93, 7.24	0.400	0.011
		Control	32	1.54	− 2.06, 5.13			
*Δ* Ommissions		VLBW	32	2.47	0.44, 4.49 *	− 3.15, 2.58	0.845	<0.001
		Control	32	2.75	0.73, 4.76 *			
*Δ* Commissions		VLBW	32	0.28	− 0.45, 1.01	− 0.141, 0.66	0.471	0.008
		Control	32	0.66	− 0.075, 1.39			

The table presents multi- and univariate results from a comparison of Not-X CPT and *Δ* Not X-CPT performance measures across young adults with VLBW and controls. Groups are matched on age, sex and years of completed education (see Table 1 for more information on demographics). MANOVA = Multivariate Analysis of variance. *Δ* = Difference score (Time epoch epoch 1). SEM = Standard error of the mean. VLBW = Very low birth weight (≤1500 g). CI = Confidence interval. *ηρ*^2^ = partial ETA squared. CPT = Continuous Performance TOT = Time on Task. RT = Reaction Time. SEM = Standard Error of the Mean.

* Indicates within-group univariate TOT effects for Δ Not X-CPT performance measures at the p < 0.05 level.

**Table 3:** Main fMRI findings: BOLD fMRI clusters (ROIs).

Anatomical region	R/L	Size in number of voxels (ROI name)	Z	Coordinates for peak activation (MNI)		
				*X*	*Y*	*Z*
**Stable task-set maintenance**						
*VLBW > Control*						
No statistically significant differences	ns	ns	ns	ns	ns	ns
*Control* > *VLBW*						
Frontal pole	L	32258 (STM ROI-1)	3.77	−21	44	20
Cingulate gyrus, anterior division	R	lm	3.56	11	36	13
Cingulate gyrus, anterior division	R	lm	3.50	3	35	0
Cingulate gyrus, anterior division	R	lm	3.49	3	37	0
Frontal pole	R	lm	3.41	15	62	−2
Frontal pole	R	lm	3.40	15	59	−4
Cingulate gyrus, posterior division	R	13596 (STM ROI-2)	3.33	6	−44	27
Cingulate gyrus, posterior division	R	lm	3.32	9	−42	27
Precuneus cortex	R	lm	3.15	12	−66	39
Cingulate gyrus, posterior division	L	lm	3.14	−3	−40	24
Cingulate gyrus, posterior division	L	lm	3.14	−3	−39	22
Cingulate gyrus, posterior division	R	lm	3.13	1	−47	34
**Adaptive task control**						
*VLBW* > *Control*						
Cingulate gyrus. posterior division	L	13173 (ATC ROI-1)	3.65	−8	−45	36
Cingulate gyrus, posterior division	R	lm	3.46	3	−38	41
Cingulate gyrus, posterior division	R	lm	3.34	2	−26	35
Cingulate gyrus, posterior division	R/L	lm	3.32	0	−42	43
Precuneus cortex	R	lm	3.26	4	−67	49
Precuneus cortex	R	lm	3.22	3	−64	40
Lateral occipital cortex, superior division	R	6180 (ATC ROI-2)	4.41	51	−59	49
Lateral occipital cortex, superior division	R	lm	4.37	52	−62	48
Lateral occipital cortex, superior division	R	lm	4.36	50	−62	49
Angular gyrus	R	lm	3.16	44	−55	59
Lateral occipital cortex, superior division	R	lm	3.08	46	−65	39
Lateral occipital cortex, superior division	R	lm	2.75	46	−71	47
*Control* > *VLBW*						
No statistically significant differences	ns	ns	ns	ns	ns	ns

Results were achieved using a mixed effects model corrected for multiple comparisons using a cluster threshold of Z > 2.3 and a corrected cluster significance threshold of p < 0.05. Main peaks and up to 5 local maxima (lm) within each cluster are reported in the table. Naming of anatomical regions was based on the Harvard Oxford cortical and subcortical structural atlases as implemented in the FSL software. Note that some clusters are relatively large and therefore span over several brain regions (see Fig. 3 and Supplementary Video 1 as well as results and discussion sections in the main text for more details). MNI = Montreal Neurological Institute, R = Right, L = Left, lm = local maxima. ROI = Region of interest (ROIs used in other analyzes). ATC = Adaptive Task Control. STM = Stable Task-set Maintenance. ns = non-significant.

**Table 4:** Between-group differences in tract average FA and MD.

Tract	Average FA (SD)		Average MD (SD)		FA t-stat (p-value)	MD t-stat (p-value)
	VLBW	Control	VLBW	Control	VLBW > Control	VLBW > Control
L ATR	0.46 (0.017)	0.47 (0.021)	7.5e-4 (2.3e-5)	7.3e-4 (1.9e-5)	ns	−3.4 (0.0014)
R ATR	0.46 (0.020)	0.47 (0.021)	7.3e-4 (2.4e-5)	7.1e-4 (1.9e-5)	ns	−2.9 (0.0047)
L CGC	0.44 (0.045	0.49 (0.027)	8.0e-4 (5.3e-5)	7.7e-4 (4.5e-5)	4.0 (0.00019)	−2.5 (0.014)
R CGC	0.41 (0.042	0.44 (0.027)	7.8e-4 (4.6e-5)	7.6e-4 (4.2e-5)	3.4 (0.00149)	ns

Multiple linear regression testing for group differences between the VLBW group and controls. Age and sex were included in the models as covariates of no interest. Results were corrected for multiple comparisons using FDR (q < 0.05). FA = fractional anisotropy. MD = mean diffusivity. SD = standard deviation. ATR = anterior thalamic radiation. CGC = cingulum. L = left. R = right. VLBW = Very Low Birth Weight (≤1500 g). ns = non-significant. CC = Cognitive Control.

**Table 5:** Within-group associations between tract average FA/MD and clinical and behavioral measures.

Analyses	Tract	FA t-stat (p-value)	MD t-stat (p-value)
**Within-group associations VLBW group**			
Birth weight*	L CGC	4.0 (0.00048)	ns
Performance-based CC	L CGC	3.5 (0.0015)	ns
	R CGC	4.0 (0.00038)	ns
Self-reported CC	L CGC	ns	−3.3 (0.0026)
Fluid Intelligence	L CGC	3.2 (0.0038)	ns
	R CGC	2.3 (0.028)	ns
**Within-group associations control group**			
	ns	ns	ns

Multiple linear regression testing for within-group effects of birth weight (BW)*, Gestational Age (GA)*, and other clinical and behavioral measures. Age and sex were included in the models as covariates of no interest. Only tracts with effects p < 0.05, with an FDR correction for multiple comparisons (q < 0.05) is shown. FA = fractional anisotropy. MD = mean diffusivity. ATR = anterior thalamic radiation. CGC = cingulum. L = left. R = right. VLBW = Very Low Birth Weight (≤1500 g). * = Associations with GA and BW were tested separately, by adjusting for the other. ns = non-significant. CC = Cognitive Control. For performance-based and self-reported CC, raw scores were inverted to allow for more intuitive interpretation (higher score = better function).

**Table 6:** Element-wise tractography in VLBW adults.

Birth weight			Self-reported CC			Performance-based CC			Anxiety			Fluid Intelligence		
	FA	MD		FA	MD		FA	MD		FA	MD		FA	MD
L ATR	-	-	L ATR	-	-	L ATR	2.8%	2.7%	L ATR	-	-	L ATR	-	-
R ATR	-	-	R ATR	-	-	R ATR	0.7%	1.0%	R ATR	-	-	R ATR	-	-
L CGC	4.0%	-	L CGC	-	-	L CGC	1.0%	-	L CGC	-	-	L CGC	0.5%	-
R CGC	-	-	R CGC	-	2.4%	R CGC	8.1%	0.6%	R CGC	-	-	R CGC	0.6%	-

The percentages of each tract showing significant associations. Analyses were corrected for multiple comparisons across all points, FA and MD, across all 5 variables tested. There were no statistically significant associations within the term-born control group. ATR = anterior thalamic radiation. CGC = cingulum. L = left. R = right. VLBW = Very Low Birth Weight (≤1500 g). ns = non-significant. CC = Cognitive Control.
