# Supplementary material for: Preterm birth leads to hyper-reactive cognitive control processing and poor white matter organization in adulthood
Source: Neuroimage. Author manuscript; Available in PMC 2019 Jul 12. (PMC6625518; doi:10.1016/j.neuroimage.2017.11.055)
Supplement: Supplementary data [file NIHMS1036642-supplement-Supplementary_data.pdf]

Supplementary Material 1. Not-X CPT task

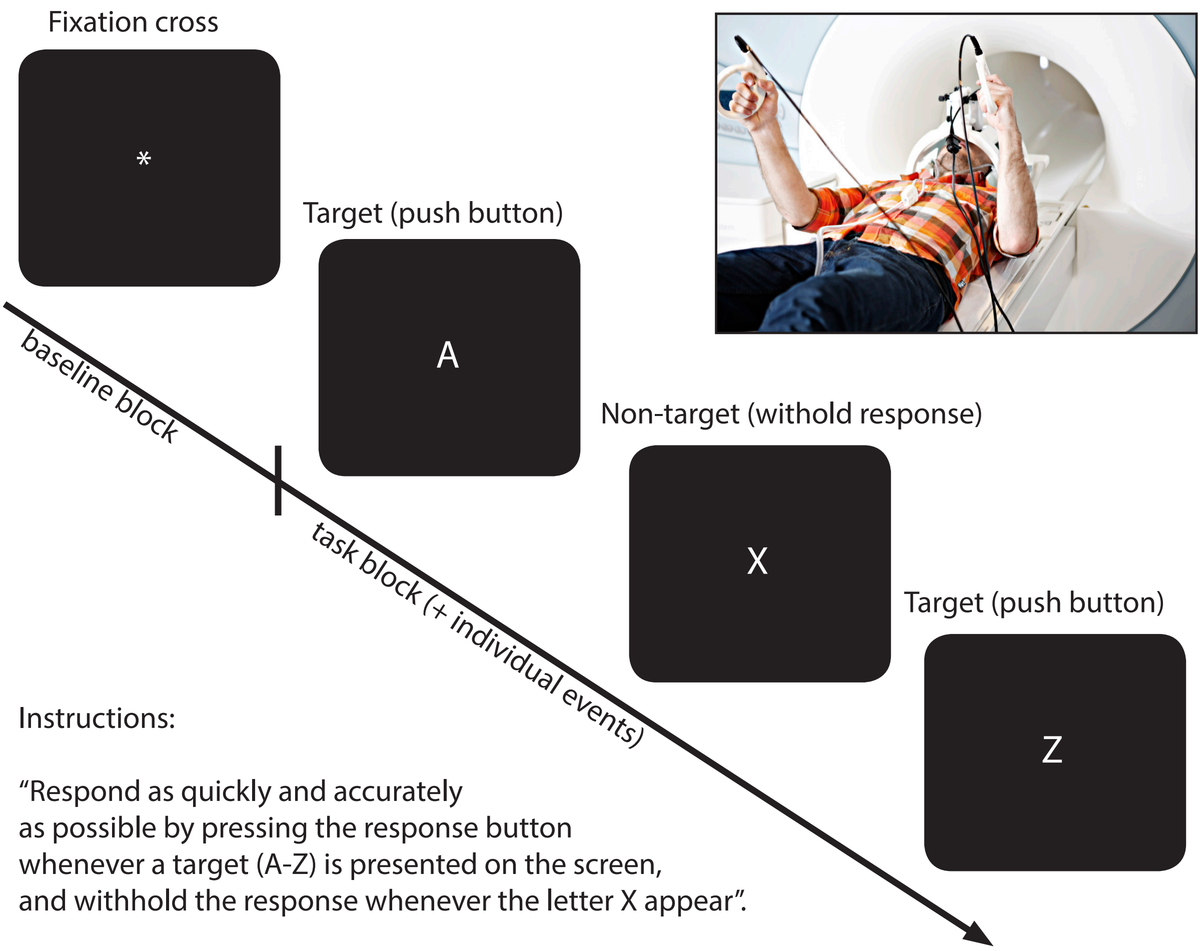

The task had 480 stimuli in total, with 432 targets consisting of randomly presented letters from A-Z other than "X", and 48 non-targets (10%), which consisted of the letter "X". Stimuli were presented on the screen for 250 milliseconds each. The task was presented to the participants in two separate runs of ~15 minutes each, consisting of 16 task blocks and 16 baseline blocks (fixation cross) each. Inter block intervals (IBIs) varied between 14, 16, and 18 seconds. Inter stimuli intervals (ISIs) varied between 1, 2, and 4 seconds, and were jittered to ensure sampling of different time-points of the hemodynamic response curve to allow for event-related fMRI analysis (Petersen and Dubis, 2012). To eliminate systematic order effects, IBIs, ISIs, and the different stimulus types (targets vs. non-targets) were counterbalanced. The design was implemented using Matlab (The Math works, Inc., Natick, USA). The two runs were combined, and two main contrasts were computed: stable task-set maintenance (STM: task block > fixation block) and adaptive task control (ATC: non-targets > targets). See Olsen et al. 2013 for more details (Olsen et al., 2013).

Supplementary Material 2. AutoMATE yields measures along white matter tracts with indices that are consistent across subjects, allowing for valid and reliable group-level analysis. Raw DTI images were first visually inspected for artifacts. Further preprocessing was performed using tools from FSL (http://fsl.fmrib.ox.ac.uk/fsl/). DTI images were corrected for eddy-current induced distortions using "eddy_correct", DWI scans were skull-stripped using BET, and Fractional anisotropy (FA) and Mean diffusivity (MD) maps were computed using "dtifit". Whole-brain DTI tractography was performed with Camino (http://cmic.cs.ucl.ac.uk/camino/). The maximum fiber turning angle was set to 40°/voxel to limit biologically implausible results, and tracing stopped when fractional anisotropy (FA) dropped below 0.2, which is the standard in the field.

As part of autoMATE, five white matter tract atlases were manually constructed from healthy young adults' (20-30 years old) HARDI data, as detailed elsewhere (Jin et al., 2014). These atlases, based on the "Eve" brain atlas (Zhang et al., 2010), included 18 major white matter tracts (Catani et al., 2007). Each subject's FA map was registered, linearly and then non-linearly, to the Eve atlas, using ANTs (Advanced Normalization Tools (Avants et al., 2011) and its ROIs were correspondingly warped to extract the 18 tracts from each subject based on a look-up table (Zhang et al., 2010). ROI registration was visually checked for all subjects, and all passed quality control. Each subject's FA map was further registered non-linearly to each of the five manually constructed atlases. All registrations were visually inspected for quality, and all passed quality control. The 18 tracts from each atlas were then warped to the subject space based on the deformation field from the registration steps (Jin et al., 2011). We refined fiber extractions of each tract based on the distance between the warped corresponding tract of each atlas and the subject's fiber candidates from ROI extraction. Individual results from the five atlases were fused. We visually inspected the resulting fiber bundles. As the resulting files include over 50,000 data points (elements per measure), we also generated tract-average measures. This allowed us to investigate both element-wise differences and more general effects. Across all the individuals in the study, we selected two example subjects (VLBW and Control) to display results of group analyses.

Supplementary Material 3. Within-group *associations of tract average FA and MD with BOLD fMRI ROI values.*

Group	BOLD-fMRI ROI	Tract	FA t-statistic (p-value)	MD t-statistic (p-value)
VLBW	Stable task-set maintenance ROI-1	R ATR	1.9 (0.032)	ns
Control				
	Adaptive task control ROI-1	R ATR	ns	1.1 (0.021)
	Stable task-set maintenance ROI-1	L ATR	2.1 (0.049)	ns
	Stable task-set maintenance ROI-1	R ATR	2.1 (0.043)	ns
	Stable task-set maintenance ROI-2	L ATR	**4.4 (0.00018)**	ns

Multiple linear regression testing for within-group effects of BOLD fMRI ROI values. Age and sex were included in the models as covariates of no interest. Only tracts with effects p <0.05 are shown. Only results highlighted in bold text survived FDR correction for multiple comparisons within variables included in this *post hoc* analysis (q<0.05). ATR = anterior thalamic radiation. FA = fractional anisotropy. MD = mean diffusivity. CGC = cingulum. L = left. R = right. VLBW = Very Low Birth Weight (≤1500 grams). ns = non-significant.

Supplementary Material 4. *Element-wise within-group associations between FA and MD, and BOLD fMRI ROI values.*

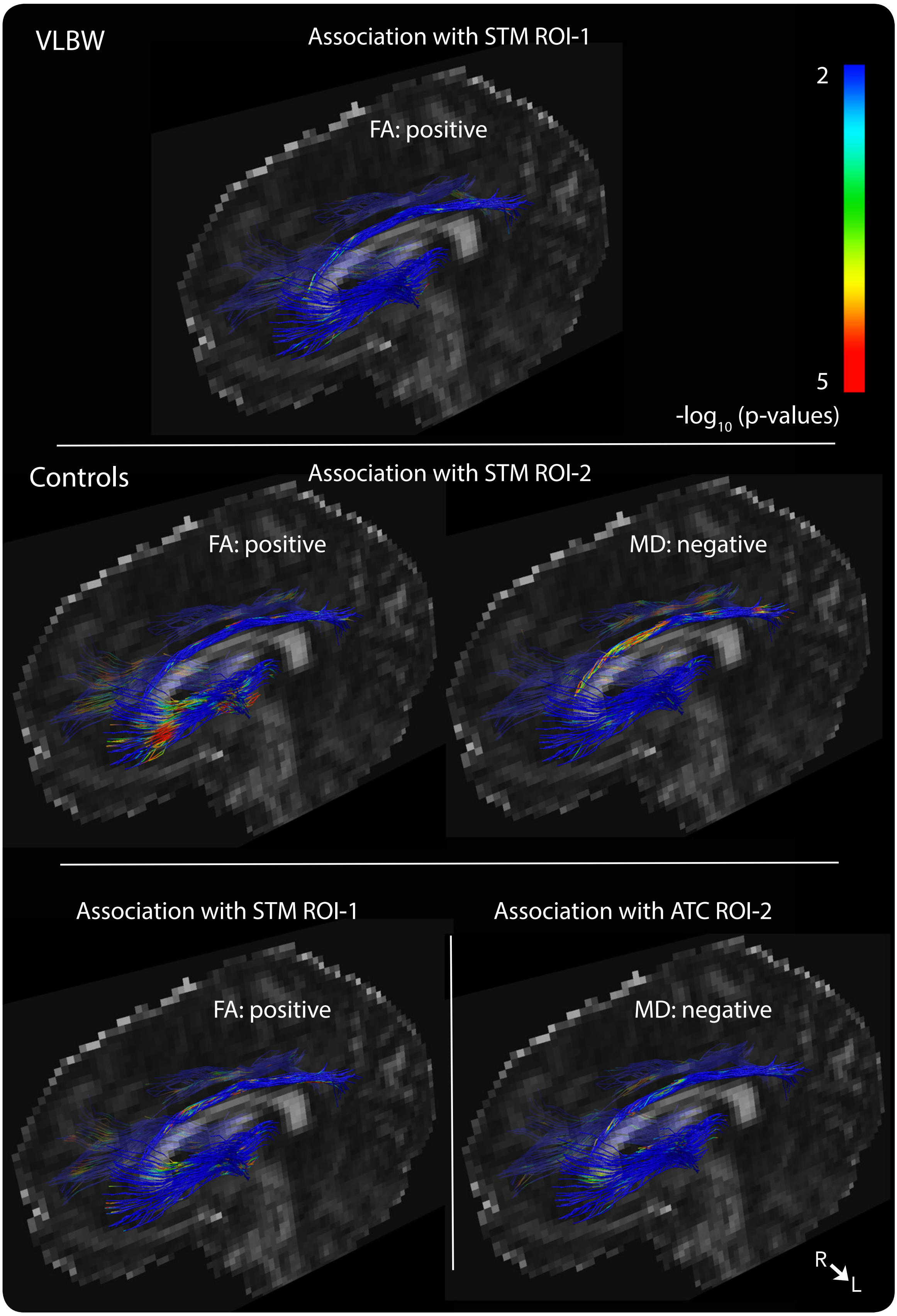

Results from a multiple linear regression analysis testing for within-group element-wise effects, co-varying for age and sex. ROI = Region of interest (ROIs used in other analyses). ATC = Adaptive Task Control. STM = Stable Task-set Maintenance. This is a thresholded p-map, with blue areas representing no significant correlation. Areas that are green-red are those showing significant correlations. Correlations are indicated as 'positive' or 'negative'. The color bar indicates the -log10(p-value). Notably, these results did not survive correction for multiple comparisons, but were included for explorative purposes.

References

Avants, B.B., Tustison, N.J., Song, G., Cook, P.A., Klein, A., Gee, J.C., 2011. A reproducible evaluation of ANTs similarity metric performance in brain image registration. Neuroimage 54, 2033-2044.

Catani, M., Allin, M.P., Husain, M., Pugliese, L., Mesulam, M.M., Murray, R.M., Jones, D.K., 2007. Symmetries in human brain language pathways correlate with verbal recall. Proc Natl Acad Sci U S A 104, 17163-17168.

Jin, Y., Shi, Y., Jahanshad, N., Aganj, I., Sapiro, G., Toga, A.W., Thompson, P.M., 2011. 3D elastic registration improves HARDI-derived fiber alignment and automated tract clustering. Biomedical Imaging: From Nano to Macro, 2011 IEEE International Symposium on. IEEE, pp. 822-826.

Jin, Y., Shi, Y., Zhan, L., Gutman, B.A., de Zubicaray, G.I., McMahon, K.L., Wright, M.J., Toga, A.W., Thompson, P.M., 2014. Automatic clustering of white matter fibers in brain diffusion MRI with an application to genetics. Neuroimage 100, 75-90.

Olsen, A., Ferenc, J., Evensen, K.A., Garzon, B., Lalandrø, N.I., Håberg, A.K., 2013. The Functional Topography and Temporal Dynamics of Overlapping and Distinct Brain Activations for Adaptive Task Control and Stable Task-set Maintenance during Performance of an fMRI-adapted Clinical Continuous Performance Task. Journal of Cognitive Neuroscience.

Petersen, S.E., Dubis, J.W., 2012. The mixed block/event-related design. NeuroImage 62, 1177-1184.
Zhang, Y., Zhang, J., Oishi, K., Faria, A.V., Jiang, H., Li, X., Akhter, K., Rosa-Neto, P., Pike, G.B., Evans, A.,

Toga, A.W., Woods, R., Mazziotta, J.C., Miller, M.I., van Zijl, P.C.M., Mori, S., 2010. Atlas-guided tract reconstruction for automated and comprehensive examination of the white matter anatomy. Neuroimage 52, 1289-1301.
